# MicroRNA: Dynamic Regulators of Macrophage Polarization and Plasticity

**DOI:** 10.3389/fimmu.2017.01062

**Published:** 2017-08-31

**Authors:** Jezrom Bokcaerin Self-Fordham, Afsar Raza Naqvi, Juhi Raju Uttamani, Varun Kulkarni, Salvador Nares

**Affiliations:** ^1^Department of Periodontics, University of Illinois at Chicago, Chicago, IL, United States

**Keywords:** microRNA, macrophage, plasticity, polarization, immune regulation

## Abstract

The ability of a healthy immune system to clear the plethora of antigens it encounters incessantly relies on the enormous plasticity displayed by the comprising cell types. Macrophages (MΦs) are crucial member of the mononuclear phagocyte system (MPS) that constantly patrol the peripheral tissues and are actively recruited to the sites of injury and infection. In tissues, infiltrating monocytes replenish MΦ. Under the guidance of the local micro-milieu, MΦ can be activated to acquire specialized functional phenotypes. Similar to T cells, functional polarization of macrophage phenotype viz., inflammatory (M1) and reparative (M2) is proposed. Equipped with diverse toll-like receptors (TLRs), these cells of the innate arm of immunity recognize and phagocytize antigens and secrete cytokines that activate the adaptive arm of the immune system and perform key roles in wound repair. Dysregulation of MΦ plasticity has been associated with various diseases and infection. MicroRNAs (miRNAs) have emerged as critical regulators of transcriptome output. Their importance in maintaining health, and their contribution toward disease, encompasses virtually all aspects of human biology. Our understanding of miRNA-mediated regulation of MΦ plasticity and polarization can be utilized to modulate functional phenotypes to counter their role in the pathogenesis of numerous disease, including cancer, autoimmunity, periodontitis, etc. Here, we provide an overview of current knowledge regarding the role of miRNA in shaping MΦ polarization and plasticity through targeting of various pathways and genes. Identification of miRNA biomarkers of diagnostic/prognostic value and their therapeutic potential by delivery of miRNA mimics or inhibitors to dynamically alter gene expression profiles *in vivo* is highlighted.

## Introduction

**Figure 1:**
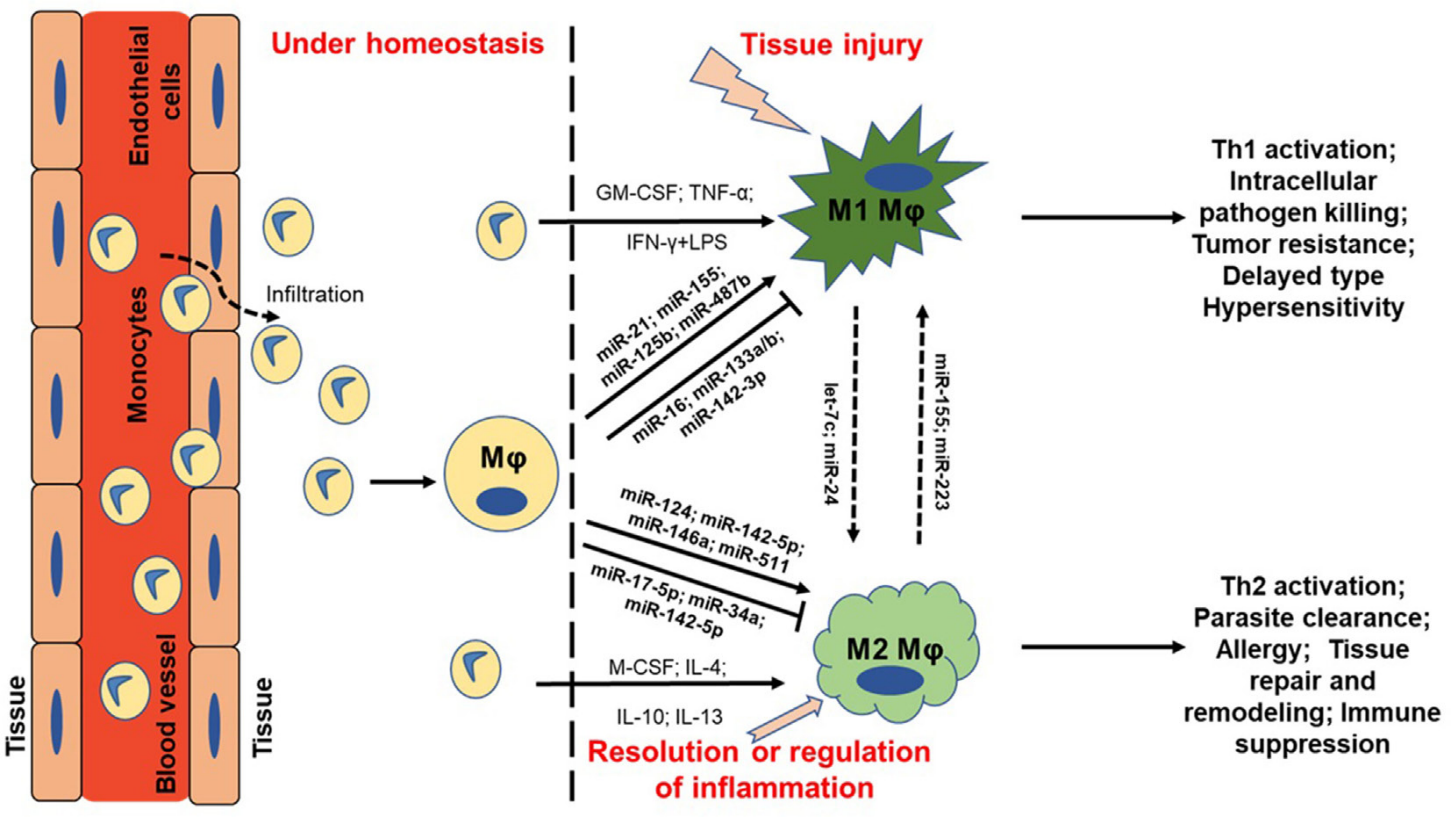
MicroRNA (miRNA) regulation of macrophage plasticity and polarization. Schematic illustration of monocyte infiltration and differentiation to macrophage (MΦ) under two distinct yet overlapping scenarios. Monocytes under the influence of proinflammatory mediators [TNF-α or IFN-γ + lipopolysaccharide (LPS)] or cytokine GM-CSF are polarized to classically activated M1 MΦ. This leads to increased expression of specific genes (M1 markers) and the cell exhibit unique phenotype, including type I inflammation, intracellular pathogen killing, and tumor resistance. Selected miRNAs are supported by empirical data in human primary MΦs with references provided in the main text. For instance, higher expression of proinflammatory miR-155 and miR-125b also favor M1 MΦ. On the other hand, cytokines (IL-4, IL-10, IL-13, or M-CSF) can polarize MΦ to a reparative (M2) type. Expression of certain miRNAs viz., miR-146a and miR-511 promotes M2 MΦ by negatively regulating genes involved in inflammatory signaling. Primary function of M2 MΦ includes Th2 activation, parasite clearance, immune suppression, and tissue repair and healing. It can be noted that M1 and M2 MΦ are considered as a continuum of two extreme rather than two distinct cell phenotypes. Hence, tissue MΦ may exhibit some features of both the described classes. Let-7c and miR-24 favors M2 phenotype while miR-155 and miR-223 can repolarize M2 MΦ toward M1 phenotype. MΦ = unstimulated MΦ. M1 = classical activation. M2 = alternative activation.

MicroRNAs (miRNAs) are short (~22 nucleotides long) non-coding RNA molecules capable of regulating gene expression at the post-transcriptional level. Since their discovery in 1993 (1, 2), our knowledge of miRNA expression and its role in health and disease has grown exponentially. Our understanding of its expression in immunity and inflammation, in particular, continues to provide new and exciting avenues for therapeutic research and clinical application. Several excellent reviews have covered miRNA functionality in the context of leukocyte differentiation (3), innate signaling (4), and T helper (Th) cell biology (5). The focus of this review are those miRNAs that have been identified as key regulators of macrophage (MΦ) polarization, the functional properties of polarized MΦs, and the ability of MΦs to switch between different activation states, i.e., regulate MΦ plasticity (Figure 1). While it is our intent to focus on MΦ polarization rather than innate activation *per se*, pathogen recognition is so deeply entwined with classical macrophage polarization that its discussion will arise on a number of occasions.

Many of the earliest studies investigating miRNA function in MΦ activation were focused on the toll-like receptor (TLR) family; most often, TLR4 and its ligand lipopolysaccharide (LPS). While acknowledging the significance of TLRs in pro-inflammatory MΦ polarization, this review will focus on the non-TLR stimuli that induce MΦ polarization. These studies not only revealed a central role for miRNA in the MΦ inflammatory response but also introduced many of the research techniques and technologies used for miRNA research today. For example, high throughput sequencing of RNA immunoprecipitated with Argonaute (Ago) proteins can identify miRNAs and targets that are part of the Ago silencing complex (6). Three different methods based on cross-linked immunoprecipitation (CLIP) followed by next generation sequencing techniques, such as HITS-CLIP, PAR-CLIP, and iCLIP, have helped make great strides toward global miRNA targets identification with higher confidence (6). The findings can be further validated by luciferase reporter assay that is commonly adopted for the purpose of predicted miRNA–mRNA target interactions.

Macrophage polarization can be pro-inflammatory or anti-inflammatory and these are commonly referred to as M1 or M2 MΦs, respectively. Along with polarization, we will also discuss how miRNA can promote or inhibit the phenomenon of plasticity viz. the transitioning of MΦs between states of opposing functionality, as well as suppress the induction or function of M1/M2 MΦs. The discovery of novel miRNAs capable of altering the choice made by MΦs facing new, usually contradictory stimuli (for example, anti-inflammatory cytokines following the removal of pathogen/pro-inflammatory stimulus), holds great therapeutic potential.

## Differentiation

In recent time, it has become clear that monocyte-to-MΦ differentiation is, in itself, a polarizing event. Two key cytokines are responsible for triggering monocyte differentiation: M-CSF and GM-CSF (7). These differentiation factors induce convergent and divergent changes in gene expression. One such study found that 87% of these changes were shared, with only 13% of the changes in gene expression being unique to M-CSF or GM-CSF (7). Truly equivalent, comprehensive miRNA profiling of human M-CSF vs. GM-CSF-mediated monocyte differentiation is, surprisingly, currently unavailable. Such data are not present in the openly accessible Gene Expression Omnibus (GEO) repository of high-throughput gene expression data, nor are they revealed by a PubMed search.

### GM-CSF and M1-Biased MΦs

GM-CSF promotes the differentiation of MΦs biased for the M1, or classical activation state. This type of MΦ is typically generated in response to a wide range of pathogens under inflammatory conditions and as itself profound pro-inflammatory. For reference, the GM-CSF signaling pathway includes janus-activated kinase (JAK), signal transducer and activator of transcription (STAT), Ras, mitogen-activated protein kinase (MAPK), and phosphoinositide 3-kinase (PI3K) molecules, all of which ultimately act to trigger NFκB activation (7). These signaling molecules are also involved in MΦ activation by pro-inflammatory and anti-inflammatory stimuli and miRNA targeting these signaling molecules (and by extension M1- vs. M2-biased differentiation) will be discussed in detail in section 3 and beyond.

To the best of our knowledge, there is no known instance of a miRNA capable of directly targeting the GM-CSF receptor. While it is likely that the GM-CSF receptor will eventually sit alongside the M-CSF receptor as a validated target of miRNA regulation, at present, we know that the production of GM-CSF is controlled by miRNA.

For example, miR-133a and miR-133b are reported to regulate GM-CSF expression (8). These data were acquired using murine cells, and while comparative human data have not been reported, complete murine–human homology for these miRNAs is suggestive of conserved functionality across species. Cross-species, or rather evolutionary, conservation of miRNA sequence and function is not uncommon. Indeed, software-based tools have been created for the specific purposes of identifying miRNA orthologs, such as microPIR2 (9). In a telling example of such conservation, Lagos-Quintana et al. searched for the human orthologs of 31 murine miRNAs and reported that only one of these was absent from the human genome (10). Furthermore, we have observed incidences where target regulation has been conserved even when miRNA sequence homology has not been identical. For example, we have extensively characterized the inhibitory effects of miR-24, miR-30b, and miR-142-3p expression on myeloid inflammatory cell viz. monocyte, MΦ, and dendritic cell (DC), functionality. Human and murine miR-24 and miR-142-3p possess 100% sequence homology, while miR-30b differs in 2 of its nucleotides. However, we have observed that the enforced expression of any of these miRNAs inhibits the production of pro-inflammatory cytokines by LPS-stimulated myeloid inflammatory cells of human (11–13) origin.

### M-CSF and M2-Biased MΦs

M-CSF promotes the differentiation of MΦs that are biased for the M2, or alternative activation, state. This type of MΦ is typically generated under normal conditions or during the resolution of inflammation and possesses potent anti-inflammatory and wound-healing properties. In reality, the dichotomy between M-CSF vs. GM-CSF mediated differentiation is likely to be blurred by the fact that monocytes are often exposed to both of these cytokines in either a simultaneous or sequential fashion. The ratio of M-CSF to GM-CSF is likely important in determining the type of MΦ that is generated, as is the influence of other pro-inflammatory and/or anti-inflammatory cytokines.

There are also intrinsic factors that influence monocyte-to-MΦ differentiation, including the type of monocyte that is being activated. Most apparently, the fact that the monocyte population is not phenotypically uniform and subsets differ in their propensity to differentiate into resting or pro-inflammatory MΦs. In human monocytes, this includes subdivision based on CD16 expression, and more recently, further subdivision based on HLA-DR expression and high/low CD14 expression (14, 15). This is another niche in which convergence/divergence in miRNA expression may provide new avenues for therapeutic research.

The M-CSF signaling pathway shares many of its intracellular components with the GM-CSF pathway, such as Ras, MAPKs, and PI3Ks. miRNAs targeting these molecules will be discussed in the context of M1/M2 polarization in section 3 and beyond. miR-22, miR-34a, and miR-155 have recently been reported to directly target the M-CSF receptor in mice (16). These miRNAs were initially reported to be upregulated during GM-CSF-mediated monocyte-to-DC differentiation. Following the identification of the M-CSF receptor as a direct target of miR-22, miR-34a, and miR-155, Riepsaame et al. further demonstrated that miRNA upregulation viz. ability to downregulate the M-CSF receptor, was required for DC maturation. This example, whereby GM-CSF antagonizes M-CSF signaling potential by increasing the expression of specific miRNA/s, provides a strong rationale/investigative justification for performing a comprehensive miRNA profiling analysis of GM-CSF vs. M-CSF-mediated differentiation of primary human monocytes.

Fontana et al. have also described miRNA-mediated downregulation of the M-CSF receptor, albeit *via* an indirect mechanism. In this study, which utilized a unilineage monocytic culture, downregulation of miR-17-5p, miR-20a and miR-106a during differentiation was associated with the upregulation of runt-related transcription factor 1 (RUNX1)—a promoter of M-CSF receptor transcription (17). Our own profiling of primary human monocyte-to-MΦ differentiation also identified the differential expression of these miRNAs and, as such, presents primary human data that are in support of their observations (11). The authors went on to identify RUNX1 as a direct target of miR-17-5p, miR-20a, and miR-106a. To demonstrate this mechanism, the authors not only showed that enforced expression of these miRNAs resulted in the downregulation of RUNX1 but also that silencing these three miRNAs resulted in its upregulation.

This study is also a prime example of a miRNA functioning as a vital component of a regulatory circuit. Here, miR-17-5p/20a/106a inhibit the translation of RUNX1 mRNA into protein, decreased levels of RUNX1 protein results in decreased transcription of the CSF1R gene, and this results in reduced responsiveness to M-CSF/M2-biased MΦ differentiation. The circuit is completed by the fact that RUNX1 inhibits the transcription of miR-17-5p/20a/106a by binding to the miRNA 17-5p-92/106a-92 cluster promoters. While this circuit appears to operate independently of M-CSF receptor ligation, other closely related cytokine receptors are likely to influence its function. For example, ligation of RANK on the monocyte/MΦ is known to downregulate RUNX1 expression (18). Following the logic of the described circuit, this may represent a means of enhancing osteoclast (OC) lineage commitment, as while M-CSF signaling promotes the early stages of monocyte-to-OC differentiation through its pro-survival properties, RANK signaling is the factor which truly differentiates between MΦ vs. OC fate.

### miRNA Expression Profiles of M-CSF vs. GM-CSF MΦs

The current literature provides limited insight into the similarities and differences in the miRNA profiles of M-CSF vs. GM-CSF-differentiated MΦs. Banerjee et al. recently investigated let-7c expression in murine bone marrow-derived MΦs (BMM) generated using M-CSF (M-BMM) or GM-CSF (GM-BMM) (19). The authors found that let-7c expression was higher in M-BMM than in GM-BMM. let-7c expression also decreased when M-BMM were converted into GM-BMM, and the opposite was true when GM-BMM were converted into M-BMM. Furthermore, in GM-BMM, overexpression of let-7c alone was sufficient to reduce the expression of M1 markers and increase M2 markers. Similarly, the silencing of let-7c in M-BMM resulted in increased M1 marker expression while reducing M2 marker expression. These data clearly demonstrate that the impact the expression of a single miRNA can have on the determination of MΦ differentiation.

Banerjee et al. also found that another miRNA, miR-125a-5p, possessed similar characteristics to let-7c (20). Their initial finding was that miR-125a-5p is expressed at a higher level in M-BMM than GM-BMM. They subsequently showed that miR-125a-5p expression was increased by TLR4/2 ligation and provided evidence that miR-125a-5p is part of the negative feedback loop that exists to reign back the pro-inflammatory potential of the innately activated MΦ. Overexpression of miR-125a-5p inhibited LPS-induced M1 marker expression while enhancing the expression of IL-4-induced M2 markers. Furthermore, the opposite was true when miR-125a-5p expression was silenced.

#### A Side Note on Multi-miRNA Regulation of Target Expression and Biological Function

As previously mentioned, evolutionary conservation of miRNA sequence and/or functionality is not uncommon. Piriyapongsa et al. also used the microPIR2 database to investigate this phenomenon. The authors found that 9 out of the 12 orthologous (murine–human) miRNA families predicted to target a specific mRNA (in this case PRKAG1) were indeed true interactions (9). This study evidences the high degree of evolutionary conservation exhibited by miRNA. It also highlights another key property of miRNA-mediated target regulation: the existence of multi-miRNA regulation of a single target. This is an understudied area of miRNA research and the paucity of data makes it difficult to infer the purpose of this phenomenon. However, it does raise a number of important questions worthy of contemplation. For example, does multi-miRNA regulation of a single target allow for greater control of target expression? Do different external stimuli, or even the strength of a single stimulus, result in a sliding scale of miRNA induction ranging from the induction of one to several regulatory miRNAs? In support of the latter, our own studies investigating LPS challenge of MΦ have revealed differential miRNA induction at different LPS concentrations (21).

Alternatively, does the existence of multiple miRNAs with common target specificity represent redundancy in the system so as to ensure operative regulation in the event of mutation? miRNAs are, after all, typically only 22 nucleotides in length. As such, the alteration of even a single nucleotide can mean the difference between targeting mRNA x or mRNA y—or no target at all. Given their susceptibility to mutation-induced changes in functionality it would certainly be advantageous for redundancy to exist and, therefore, for the multi-miRNA approach to target regulation to be subject to positive selective pressure. In our own study of three miRNAs (miR-24, miR-30b, and miR-142-3p) whose expression were downregulated during MΦ differentiation and in response to LPS, and whose inhibitory potential are comparable, we have not observed synergy in their action (14). Which is to say that the enforced expression of all three had no greater effect than that conferred by the most potent of the three (miR-142-3p). This observation may be hypothesized to be the result of redundancy, at least as far as the anti-inflammatory potential of these three miRNAs is concerned.

These factors, along with other compounding factors such as indirect target regulation, coordinated regulation of specific biological processes by multiple miRNAs at the network level, and non-canonical modes of miRNA action (for example, increased mRNA translation *via* increased mRNA stability upon miRNA-binding) indicate that our understanding of miRNA is still in its infancy. While this complexity may introduce “speed bumps” for researchers looking for a quick clinical application for their findings, it also serves to emphasize the tremendous therapeutic potential of manipulating miRNA expression to treat disease.

#### A Side Note on miRNA Regulation of Monocyte/MΦ Survival

With the aim of providing a biological rationale for miRNA-mediated regulation of the M-CSF receptor, we will briefly discuss its biological relevance.

First, receptor-mediated desensitization to extracellular ligands is a common way of preventing over-stimulation. With regard to M-CSF and NF-κB activation, the fact that NF-κB possesses both pro-apoptotic and anti-apoptotic properties provides opportunities for dysregulated activation to result in cell death or cell transformation (22). With this in mind, we turn to the observation that M-CSF receptor degradation is greater in mature MΦs, which are long-lived than in monocytes, which are programed to undergo apoptosis in the absence of anti-apoptotic signaling, such as is provided by M-CSF (23, 24). There is an obvious advantage to the monocyte of prolonging M-CSF signaling viz. the expression of NF-κB induced anti-apoptotic genes, such as BCL2, BCL2L1, and BCL2A1 (25). Conversely, the mature MΦ would benefit from reduced M-CSF signaling—and associated NF-κB activation—as this would set a threshold that allows the MΦ to respond to a plethora of NF-κB-activating danger signals. The ubiquitous nature of NF-κB activating signals, which include many TLR ligands and many soluble mediators of inflammation, means that this threshold is of central importance to MΦ biology—from their generation to function and fate.

Our investigation of M-CSF-mediated monocyte-to-MΦ differentiation has revealed a number of miRNAs whose pattern of expression and functionality likely contribute to the setting of this threshold (11). These miRNAs, which include miR-26a and miR-142-3p, are pro-apoptotic as their targets include anti-apoptotic genes, for example, members of the BCL2 family (11). As polarized MΦs are associated with certain microenvironments, for example, inflammation is associated with pro-apoptotic cytokines, such as TNF-α (26), and wound healing is associated with anti-apoptotic cytokines such as IL-10 (27), it will be interesting to see whether these miRNAs regulate MΦ survival in a manner that is independent of (or dependent upon) MΦ polarization state. Equally, while we have observed the regulation of survival by these miRNAs on M-CSF differentiated MΦs, we are yet to investigate whether this is true in GM-CSF-differentiated MΦs. If their ability to regulate survival does prove to be different for M-CSF vs. GM-CSF-differentiated cytokines, then these miRNAs could, in theory, be used to regulate the M1/M2 balance *via* disproportionate deletion.

## Pro-Inflammatory Polarization

### Classical Activation and M1 MΦs: Initiating and Propagating Inflammation

M1, or classical, activation can be induced by a combination of IFN-γ and TNF-α. While LPS is also commonly used, with or without IFN-γ priming, this more accurately represents a combination of innate activation (LPS) and classical activation (IFN-γ plus autocrine TNF-α). For the purpose of this review, we will consider IFN-γ and TNF-α, along with other pro-inflammatory cytokines such as IL-1β, as the key mediators of the pro-inflammatory M1 MΦ. A number of excellent reviews that have previously covered miRNA regulation of innate MΦ activation are available to the reader (4, 28, 29). In this section, we highlight some key examples of miRNAs involved in generating the M1 MΦ. Table 1 enlists miRNAs and the targeted pathways/genes through which they regulate the M1 phenotype.

**Table 1:** Key microRNA (miRNAs) involved in M1 (GM-CSF) MΦ differentiation and modulation of pro-inflammatory polarization.

miRNA	Function	Reference
miR-133a, miR-133b	Regulate GM-CSF expression in murine cells	(8)
miR-3473b	Downregulated by INF-γ that increases phosphatase and tensin homolog expression; suppressing Akt-signaling and IL-10	(31)
miR-132, miR-26a	Upregulated upon *Mycobacterium tuberculosis* infection; inhibits IFN-γ signaling	(32)
miR-155, miR-146a	Negative regulators of NO production	(34, 35)
miR-144, miR-155, miR-146a, miR-145, miR-222, miR-27a, miR-27b	Involved in the macrophage response to *M. tuberculosis* infection	(40–42)
miR-181a	Direct regulation of TNF-α production	(46)
miR-146a, miR-142-3p	Indirect regulation by targeting multiple components of TNF-α signaling pathway (e.g., MyD88, IRAK1, TRAF6, TLR4/2)	(47–52)
miR-146a	Decreases IL-6 production by targeting NOTCH1	
miR-223	Downregulation of miR-223 increases STAT3, thus increasing IL-6 production	(56)
let-7b	Mediates IL-6 regulation by a microvesicle (MV)-based mode of inhibition	(57)
miR-487b	Regulates IL-33 expression, which induces TNF-α and enhances antigen-presenting cell (APC) functionality	(61)
miR-16	Targets IL-12(p40) as demonstrated in an animal model of colitis	(67)
miR-142-3p	Decreases bone resorption by suppressing M1 MΦ activation and inhibiting their conversion into OC	(69)
miR-615-3p	Targets ligand-dependent nuclear receptor corepressor (corepressor of PPARγ) and enhances MΦ phagocytosis	(72)
miR-15a/16 and miR-24/30b/142-3p	Inhibit MΦ phagocytosis	(13, 71, 77)

### IFN-γ

IFN-γ is commonly thought of as an activating and/or priming signal for the M1 MΦ. We will first consider it as an activating signal in and of itself. We have long known that IFN-γ induces many changes in MΦ gene expression at the level of both transcription and translation (30). We now know that alterations in miRNA expression are another important mediator of its phenotypical effects. For example, Wu et al. have reported that miR-3473b is downregulated by IFN-γ and demonstrated that its downregulation is required for normal priming to occur (31). The authors identified phosphatase and tensin homolog (PTEN) as a direct target of miR-3473b. PTEN is a molecule that inhibits Akt signaling and IL-10 production. In this regulatory circuit, IFN-γ signaling suppresses the ability of MΦs to produce IL-10 *via* a mechanism that involves decreased miR-3473b expression, which results in increased PTEN expression, which in turn results in decreased Akt signaling and IL-10 production.

Experiments using *Mycobacterium tuberculosis* (*M. tuberculosis*) have revealed a number of IFN-γ associated miRNAs. miR-132 and miR-26a were among 31 miRNAs identified by Ni et al. as differentially expressed in primary human MΦs infected with *M. tuberculosis* (32). miR-132 and miR-26a, which were upregulated upon infection, were found to be negative regulators of transcription coactivator p300. p300 is part of the IFN-γ signaling cascade, which means that miR-132 and miR-26a are inhibitors of IFN-γ induced signaling. Induction of these miRNAs may be added to the list of mechanisms allowing *M. tuberculosis* to survive in what would normally be considered to be the highly hazardous environment of the M1 MΦ.

miR-155 is another miRNA that is linked to IFN-γ signaling and the anti-bacterial MΦ response. The production of nitric oxide (NO) is an important component of the IFN-γ induced MΦ response (33). Data on miRNA regulation of murine MΦ NO production include two familiar faces in the miRNA realm: miR-155 and miR-146a. Qin et al. recently identified miR-155 as a negative regulator of IFN-γ induced NO production in murine MΦs (34). miR-155 achieves this by targeting CCAAT/enhancer binding protein β (C/EBPβ)—a positive transcriptional regulator of nitric oxide synthase (NOS2). Meanwhile, Li et al. showed that miR-146a expression promotes mycobacterial survival in MΦs (35). Here, miR-146a suppresses NO production by inhibiting the NF-κB and MAPKs pathways responsible for upregulating the NOS2 gene.

The importance of NO production by human MΦs is contentious. Early experiments focused on molecules proven to induce NO production in murine MΦ; however, neither LPS nor IFN-γ nor TNF-α elicited the production of NO in human MΦs (36). Still, this negative *in vitro* data did not agree with *in vivo* data identifying elevated NO levels during infection and/or inflammation (37, 38). This leads to the notion that the human immune system may have evolved alternative mechanisms for achieving the same anti-bacterial effect. Over the last decade, it has become clear that NO production is indeed an important component of the anti-bacterial activity of human MΦs, and this has been accompanied by the identification of inducers of NO, such as surfactant protein A (SP-A) (39). To the best of our knowledge, miRNAs capable of regulating the production of NO by human MΦs have yet to be identified.

Publications exploring the role of miRNA in human *M. tuberculosis* infection are scarce. A PubMed search for “tuberculosis + microRNA + MΦ” identified 52 publications, only 4 of which utilized primary human MΦs and *M. tuberculosis*, and these were published in the last 5 years. However, the following miRNAs are known to be involved in the MΦ response to mycobacterial infection: miR-144 (40), miR-132 (32), miR-26a (32), miR-155 (41), miR-146a (41), miR-145 (41), miR-222 (41), miR-27a (41), miR-27b (41), and miR-125b (42). The reader is provided with references for recent reviews on this subject for greater detail (43, 44). Interestingly, a recent publication in murine MΦs identified miR-142-3p as a regulator of the pro-inflammatory cytokine response to *Mycobacterium bovis* (45). Our studies on miR-142-3p have revealed human–murine functional homology in the context of bacterial-induced pro-inflammatory cytokine production (11, 13). It will be interesting to investigate the impact this miRNA has on the infection of human MΦs by mycobacteria, as we have also shown this miRNA to inhibit bacterial phagocytosis (13).

### TNF-α

The production of TNF-α by MΦs is regulated by several miRNAs *via* a combination of direct regulation [e.g., miR-181a (46)] and indirect regulation. The indirect regulation includes miRNA targeting of associated surface receptors, intracellular signaling molecules, and components of protein trafficking/secretion machinery. Many of these indirect mechanisms of suppression involve the targeting of multiple components of a signaling pathway by a single miRNA. For example, miR-146a regulates not only TLR4 expression but also the downstream signaling molecules MyD88, IRAK1, and TRAF6 (47, 48). These signaling molecules are utilized by other receptors associated with TNF-α, for example, TLR2 (49–51), and as such, upregulation of a miRNA (i.e., miR-146a) may inhibit MΦ activation across a wide range of pro-inflammatory stimuli. Our own work on miR-142-3p has revealed an inhibitory mechanism for this miRNA, which, in addition to the suppression of TLR4/2 mediated signaling, involves the accumulation of TNF-α at the MΦ cell surface. This phenotype arises from the known role for miR-142-3p in regulating cytoskeletal rearrangement (14, 52).

### IL-6

miR-146a has also been shown to regulate MΦ IL-6 production (53). This is likely to involve the targeting of Notch1—an inducer of IL-6 production—as it is a predicted target whose expression was reported to be decreased at the level of mRNA and protein when miR-146a expression was enforced. However, evidence for direct miRNA–mRNA binding was not included in this study. An example of a miRNA that does directly regulate IL-6 is miR-181b (54). This miRNA is upregulated in MΦs upon LPS stimulation and this response is required for the induction of IL-6 tolerance.

The production of IL-6 by MΦs is also inhibited by miR-142-3p (11, 13); however, unlike TNF-α, this is not associated with sequestration at the cell surface. Our own studies suggest that this is indirect regulation—most likely involving the targeting of one or more of the intracellular signaling molecules present between TLR4 and NF-κB (14). This is also an area where convergent miRNA regulation appears to exist, as these same studies identified a very similar phenotype for miR-24 and miR-30b to that of miR-142-3p. These three miRNAs are downregulated during monocyte-to-MΦ differentiation (11) and their enforced expression in mature MΦs inhibits NF-κB activation and cytokine production (11–13). Bioinformatic analysis revealed multiple distinct and overlapping targets associated with the TLR4-NF-κB signaling pathway. While our findings revealed the inhibitory capacity of miR-24/30b/142-3p mimics on MΦ cytokine production, the use of corresponding miRNA inhibitors did not reveal any enhancement of cytokine production. However, evidence for this reciprocal result was recently described by Liu et al. (55). Here, miR-142-3p expression in MΦs was shown to decline with age and this contributed to increased IL-6 production.

Downregulation of miR-223 expression in MΦ has also been reported to increase IL-6 production (56). This decrease resulted in increased STAT3 expression, as STAT3 is a direct target of miR-223. Interestingly, the production of IL-1β, but not TNF-α, was similarly enhanced. This convergence/divergence is mirrored by our studies on miR-24, miR-30b and miR-142-3p mediated cytokine regulation. Here, we found that TNF-α, IL-6, and IL-12p40, but not IL-8 nor IL-10, were inhibited by the introduction of miRNA mimics. The ability to manipulate the cytokine profile of MΦ rather than a single cytokine, *via* the introduction of a single miRNA mimic/inhibitor, may confer greater therapeutic potential.

**Figure 2:**
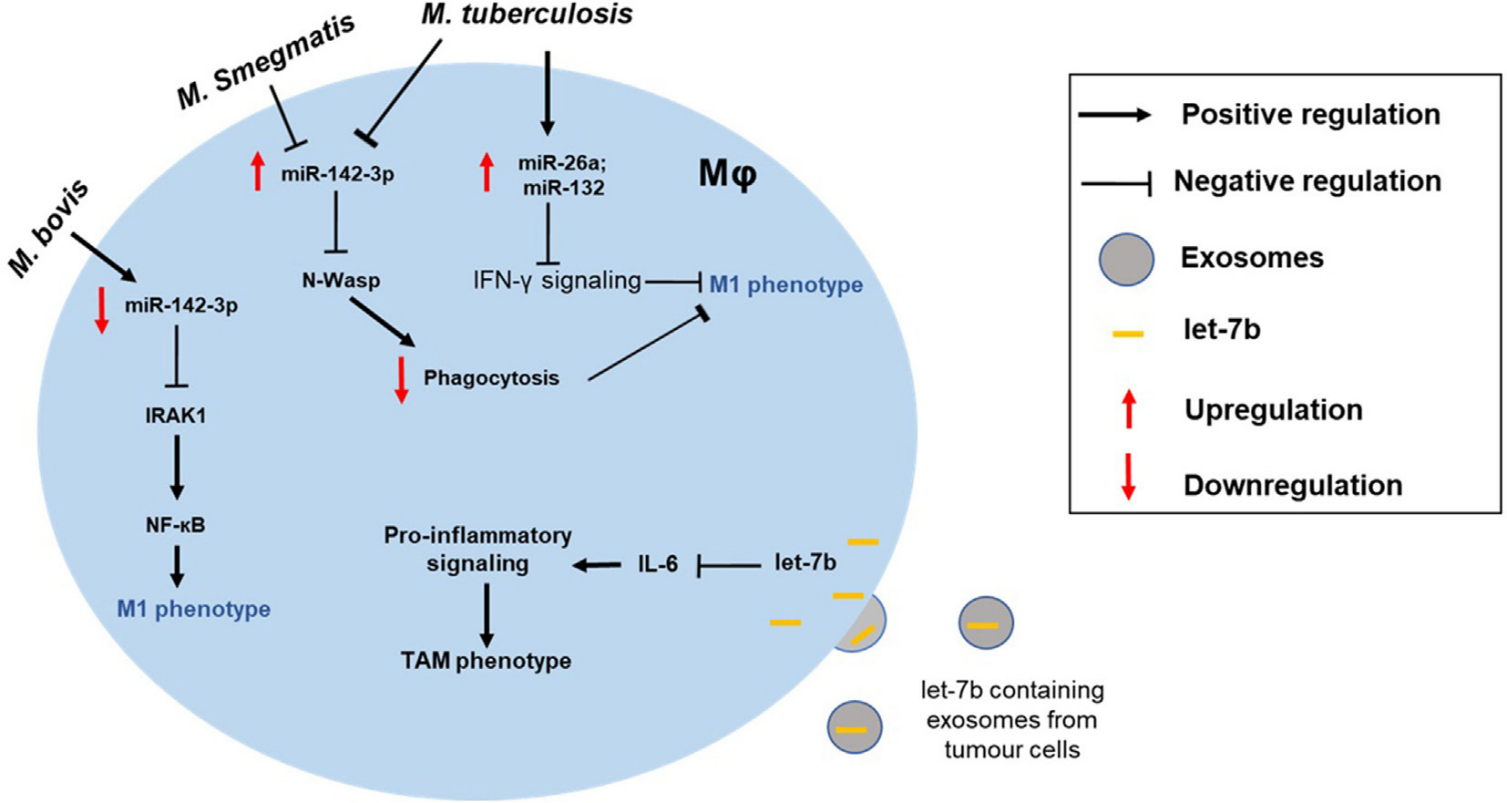
Altered macrophage polarization can contribute to the disease pathogenesis. MΦ phenotype can be skewed toward either type and can manifest disease progression. For instance, the intracellular pathogen *Mycobacterium tuberculosis* can modulate expression of miRNAs (e.g., miR-26a and miR-132) to block IFN-γ signaling in M1 MΦ. Differential impact of mycobacterial strains on MΦ is also shown. While *M. tuberculosis* and *Mycobacterium smegmatis* upregulate miR-142-3p and suppress M1 phenotype, *Mycobacterium bovis* infection leads to repression of miR-142-3p and, hence, increased NF-κB signaling through derepression of IRAK1. Exosome-mediated delivery of miRNAs can also modulate MΦ polarization of tumor-associated MΦs (TAMs). For instance, uptake of let-7b by tumor MΦ blocks IL-6 signaling skewing them toward M2-like phenotype.

Lastly, an interesting example of miRNA-mediated IL-6 regulation was recently reported by Li et al., who described a microvesicle (MV)-based mode of inhibition (57). In this case, the miRNA was let-7b and was noted to be released in MV-packaged form by LPS-stimulated tumor cells. These MVs were taken up by tumor-associated MΦs (TAMs) resulting in their acquisition of let-7b (Figure 2). Expression of let-7b then reduced MΦ IL-6 expression. The topic of MV-packaged miRNAs, from their detection as biomarkers of disease to their potential as vehicles for the therapeutic delivery of miRNA mimics/inhibitors *in vivo*, is an exciting one and is covered in detail elsewhere (58).

### IL-33

IL-33 is a novel IL-1-like cytokine simultaneously identified by Dinarello (59) and Schmitz et al. in 2005 (60). IL-33 is a regulator of MΦ activation and its expression is modulated by miR-487b (61). IL-33 promotes activation in a bipartisan manner: on the one hand, it promotes the generation of M2 MΦs (62), but on the other hand, it can induce the production of pro-inflammatory mediators, such as TNF-α (55). It can also enhance the antigen-presenting cell (APC) functionality of MΦs by increasing MHC I, MHC II, CD80, and CD86 expression (61). The significance of the IL-33/miR-487b axis is emphasized by the ubiquitous nature of its impact: this is a regulatory unit that is present during MΦ differentiation, promotes both M1 and M2 activation, and, by virtue of its role in APC function, exists at the interface between innate and adaptive immunity. Our studies have revealed a similar, but less pronounced, impact on these same functions for miR-24/30b/142-3p. By comparison, these three miRNAs also influence MΦ differentiation (11), regulate M1 vs. M2 activation (11–13), and negatively impact APC functionality (63).

### IL-12

IL-12 is a pro-inflammatory cytokine produced by MΦs in response to bacterial infection (64). The IL-12 family includes IL-12, IL-27, and IL-23. These share homology at the subunit, receptor, and signaling level (64). They are primarily pro-inflammatory by virtue of their support for Th1 (IL-12 and IL-27) and Th17 (IL-23) differentiation (65), but can also activate MΦs directly. For example, IL-12 in conjunction with IL-18 induces autocrine IFN-γ signaling in MΦs (66). We have previously reported that enforced expression of miR-24, miR-30b, or miR-142-3p in activated MΦs inhibits their production of IL-12(p40) (11, 13). p40 is the subunit shared by IL-12 (composed of p35 and p40 subunits) and IL-23 (composed of p19 and p40 subunits). Although we did not specifically look at the levels of IL-12 vs. IL-23 in these experiments, our subsequent studies suggest that this deficit in IL-12p40 (viz. IL-12 and IL-23) negatively impacts the generation of Th1 and Th17 cells. This is discussed further in section 7.

miR-16 also targets IL-12(p40) (67). It is also one of the few miRNAs whose therapeutic potential has been demonstrated in an animal model of inflammatory disease-colitis (67). miR-21 is another miRNA implicated in the regulation of IL-12. Although this evidence comes from DC rather than MΦs, the finding that miR-21-deficient (murine) DC produce less IL-12 in response to LPS is worth investigating in MΦs.

### A Side Note on Bone Resorption and M1 MΦs

Macrophages contribute to bone regeneration by several means, including the production of key cytokines involved in the differentiation of OC and osteoblasts. This facet of their functionality is often overshadowed by their key role in pathogen recognition and removal, despite the considerable social and economic costs associated with disorders of bone regeneration. They are important players in bone homeostasis, contribute to the regeneration of damaged/broken bones, and may exacerbate—or limit—the pathology of bone diseases, such as osteoporosis, rheumatoid arthritis, and diabetes-associated alveolar bone destruction (68). In these examples, the M1 MΦ can enhance pathology through their copious production of pro-inflammatory cytokines, chemokines, and metalloproteinases. These factors enhance the infiltration of damaging leukocytes, cause degradation of the extracellular matrix and promote the generation of bone-resorbing OCs. With regard to OCs, MΦs are doubly important, as not only do they secrete factors that promote the differentiation and bone-resorbing capacity of OCs, but they can also fuse with other myeloid inflammatory cells (*viz*. monocytes, DCs, and other MΦs) to form OCs. This phenomenon presents a pathway that links MΦ pathogen recognition to changes in bone regeneration. We have reported that miR-142-3p, in addition to suppressing the generation and activation of M1 MΦs, also inhibits their conversion into OC (69).

### Phagocytosis and M1 MΦs

Phagocytosis is a key function of MΦs. Phagocytosis can occur *via* a range of mechanisms, and while each of these ultimately results in the internalization of external material, each mechanism is also accompanied by unique activating stimuli. The clearest example of this would be the difference between the phagocytosis of bacteria (which is typically pro-inflammatory) vs. the uptake of apoptotic cells (which is typically anti-inflammatory) (70).

MicroRNAs regulation of phagocytosis has been reported by several groups, including ourselves (14, 71), and extends from regulation of the process itself through to nuances in concomitant activation. miR-615-3p enhances the phagocytic capacity of MΦs by targeting ligand-dependent nuclear receptor corepressor (LCoR), which is a corepressor of peroxisome proliferator-activated receptor γ (PPARγ) (72). As PPARγ enhances phagocytosis (73), the indirect effect of miR-615-3p expression is to enhance the phagocytic capacity of MΦs. It should be noted that the role of PPARγ in governing MΦ function is not limited to phagocytosis and extends to promoting alternative activation (74), differentiation (75), and lipid metabolism/foam cell formation (76).

miR-15a/16 is a miRNA cluster with the capacity to inhibit phagocytosis along with several related activation pathways (77). Originally identified as a miRNA that is upregulated by bacterial infection, the authors found that MΦs generated from miR-15a/16−/− mice possessed greater phagocytic capacity than their wild-type (WT) counterparts. Furthermore, the phagocytic capacity of WT MΦs was reduced when miR-15a/16 expression was enforced. Additional characterization of miR-15a/16−/− MΦs revealed a complex set of mechanisms responsible for the miR-15a/16-induced phenotype. These involved changes in TLR4 expression as well as modulation of downstream signaling molecules.

We have previously described miR-24, miR-30b, and miR-142-3p-mediated regulation of MΦ phagocytosis (13, 71). All three of these miRNAs were originally identified by our profiling of human monocyte-to-MΦ differentiation (11) and have proven to be regulators of many key functions of not only MΦs but also monocytes and monocyte-derived DC. The phenotypic effect of these miRNAs is the same with regard to phagocytosis but diverges with regard to the underlying mechanism. We have observed defects in cytoskeletal rearrangement—a basic physical requirement of phagocytosis—when expression of any of these miRNAs is enforced in MΦs. While the effect of miR-142-3p appears to be mediated, at least in part, through its direct targeting of PKCα, neither miR-24 nor miR-30b directly targets PKCα. Similarly, while the pro-inflammatory cytokine response to bacterial phagocytosis is inhibited by enforced expression of all three miRNAs, differences in the magnitude of the effect suggest that they may target different components of a common pathway.

## Anti-Inflammatory Polarization

### Alternative Activation and M2 MΦs: Resolving Inflammation

Alternative activation in MΦs is primarily mediated by IL-4 and IL-13 (28). A recent study by Su et al. (78) is worth highlighting for its rigorous approach that evolved from initial identification of miRNA–mRNA target interaction and function through to a therapeutic phase that included miRNA-based treatment of an animal model of disease. Furthermore, this study was supported by human data where miRNA expression and disease correlated with their *in vitro* and *in vivo* data. Such an approach should be the gold standard for miRNA-focused studies; certainly, those whose ultimate goal is a therapeutic application. A list of miRNAs involved in M2 MΦ skewing or inhibition is provided in Table 2 along with the targeted pathways/genes.

**Table 2:** Key microRNAs (miRNAs) involved in M2 (M-CSF) MΦ differentiation and modulation of anti-inflammatory polarization.

miRNA	Function	Reference
miR-22, miR-34a, miR-155	Direct target of M-CSF receptor in mice	(16)
miR-17-5p/20a/106a	Acts in a regulatory circuit suppressing runt-related transcription factor 1 translation, leading to decreased CSF1R gene transcription, which results in reduced M-CSF/M2-biased MΦ differentiation	(17)
let-7c, miR-125a-5p	Expression is higher in M-CSF-derived MΦs as compared to GM-CSF derived	(19, 20)
miR-142-5p, miR-130a-3p	Contributors to the pro-fibrogenic MΦ program	(78)
miR-511	Upregulated in M2 MΦs and downregulated in M1 MΦs both *in vitro* and *in vivo*	(80)
miR-124	Upregulated by M2 markers IL-4 and IL-13	(81, 82)
miR-23a/27a/24-5p	Downregulated in TAMs; overexpression was capable of suppressing tumor growth *in vivo*	(85)
miR-155	Enhances pro-inflammatory MΦ phenotype and attenuates the effects of TGF-β	(88)
miR-21	Inhibits PGE2 mediated M2 MΦ polarization	(96)
miR-146b	Suppresses ADA2 expression in human MΦs and inhibits pro-inflammatory cytokine release	(100)
miR-16	Regulates MΦ activation by targeting adenosine receptor A2a	(101)
miR-483, miR-877, miR-337-5p, miR-546 and miR-494 are upregulated, and miR-770-5p, miR-487b, miR-220, miR-212 and miR-712 as down-regulated	Regulated by adenosine signaling in M2 MΦs	(102)
miR-24, miR-30b, miR-142-3p	Downregulated during monocyte-to-MΦ differentiation. Their enforced expression inhibits NF-κB activation and cytokine production in mature MΦs	(11–13)

Su et al. investigated the miRNA profiles of alternatively activated MΦs and identified miR-142-5p and miR-130a-3p as important contributors to the pro-fibrogenic MΦ program (78). This program was sustained by a two-pronged mechanism that included miRNA upregulation—and resultant target suppression, and miRNA downregulation—and the resultant increase in target expression. Increased miR-142-5p expression resulted in decreased SOCS1 expression, while a decrease in miR-130a-5p expression resulted in increased PPARγ expression. The combined effect of these changes was an increase in STAT6-mediated signaling, as SOCS1 is a negative regulator of STAT6 phosphorylation while PPARγ coordinates STAT6 signaling. The authors provided additional data regarding the clinical relevance and potential therapeutic importance of this regulatory process. Mirroring their *in vitro* results, analysis of MΦ miR-142-5p and miR-130a-5p expression in tissue samples from patients with liver cirrhosis or idiopathic pulmonary fibrosis revealed the same pattern of miRNA-target expression. By using mouse models of fibrosis, they were also able to demonstrate therapeutic efficacy *via* the introduction of miR-142-5p inhibitor and miR-130a-3p mimic. This was true for both CCL4-induced liver fibrosis and bleomycin-induced lung fibrosis.

### IL-4 and IL-13

These two cytokines are functionally very similar due to their mutual capacity to trigger IL-4 receptor-mediated signaling (79). Despite this redundancy, many *in vitro* protocols utilize both, as their production and function in the context of immunity are fundamentally interconnected.

miR-511 is associated with IL-4 and IL-13 induction of the M2 MΦ. It is a miRNA that Karo-Atar et al. identified as being upregulated in M2 MΦs and downregulated in M1 MΦs both *in vitro* and *in vivo* (80). This study did not validate any specific targets for miR-511; however, bioinformatic analysis identified a number of predicted targets involved in wound healing responses and inflammation. Another miRNA, which promotes M2 MΦs, is miR-124. This miRNA was first identified by Ponomarev et al. in the context of microglia, where its phenotypical effects were associated with the suppression of experimental autoimmune encephalomyelitis (EAE) (81). Subsequent studies characterized this miRNA as being upregulated by IL-4 and IL-13. Furthermore, when IL-4/IL-13-induced miR-124 expression was inhibited *via* the introduction of an inhibitor, the expected upregulation of M2 markers (and downregulation of M1 markers) was diminished (82).

Czimmerer et al. profiled miRNA expression in IL-4-mediated activation and identified miR-342-3p, miR-99b, and miR-125a-5p as regulators of MΦ survival (83). These miRNAs are involved in IL-4/STAT6 signaling, with miR-342-3p directly targeting an anti-apoptotic network that includes BCL2L1. Another recently identified miRNA associated with M2 MΦs is miR-720 (84). This miRNA suppresses M2 activation by directly binding to GATA binding protein 3 (GATA3). Its expression is downregulated in both M2 MΦs and the functionally similar TAM. These TAMs were isolated from breast cancer patients, thus providing valuable *in vivo* context to the functioning of these miRNAs. The miR-23a/27a/24-5p cluster has also been shown to be downregulated in TAMs (85). Ma et al. described a feedback loop mediated by this cluster capable of regulating cancer progression (85). At the MΦ level, these miRNAs promote M1 over M2 polarization *via* the suppression of M2 MΦs through distinct, but related mechanisms. For example, miR-23a suppresses the JAK1/STAT6 pathway by directly targeting these molecules, while miR-27a directly targets IRF4 and PPAR-γ. In an important test of potential clinical application, the authors also demonstrated that overexpression of the miR-23a/27a/24-5p cluster was capable of suppressing tumor growth *in vivo*.

### TGF-β

TGF-β is a multifunctional cytokine with a significant role in the wound-healing process—which includes the generation of the M2 MΦ (86). TGF-β signaling may also be thought of as an inhibitory signal as it possesses a range of immunosuppressive and anti-inflammatory properties (87). However, as these effects are largely context-dependent (viz. affected by the presence of other cues in the microenvironment), we will focus on the relationship between miRNA, TGF-β, and the M2 MΦ. Here, we highlight the TGF-β associated effects of two miRNAs that have been widely reported in the context of MΦ polarization: miR-155 and miR-29b.

In light of its pro-inflammatory potential, it is not surprising that miR-155, being one of the most potent enhancers of the pro-inflammatory MΦ phenotype, should act to attenuate the effects of TGF-β (88). This occurs *via* its targeting of SMAD2—one of the key intracellular components of the TGF-β signaling pathway. miRNA biogenesis is also regulated by TGF-β/SMADs and, as such, appears to represent an important autoregulatory feedback loop. miR-29b is another miRNA whose expression in MΦs has been reported to be upregulated by TGF-β. Furthermore, this has been linked to the defective functioning of MΦs after bone marrow transplantation (89). Here, miR-29b upregulates COX-2 expression *via* indirect means resulting in MΦs with lesser bactericidal activity. In addition to providing evidence for miR-29b upregulation in transplantation patients, the authors also demonstrated that transfection with a miR-29b inhibitor restored the bactericidal activity of these MΦs.

### PGE2

Alternative activation can also be induced by stimulating MΦs with prostaglandins (90). The functional effects of prostaglandins are, however, closely connected to other stimuli relevant to MΦ activation, such as corticosteroids (91) and adenosine (92). For example, corticosteroids enhance adenosine receptor signaling (93), adenosine and PGE2 signaling is synergistic (94), and PGE2 secretion is regulated by corticosteroids (95). This is an under-developed area of miRNA research and as such, limited data are available; however, a number of relevant miRNAs have been discovered.

miR-21 is a regulator of PGE2-mediated MΦ polarization. A recent study found that PGE2 decreases miR-21 expression in MΦs and that this contributes to the expression of genes associated with M2 MΦs (96). Furthermore, when the authors enforced miR-21 expression, it prevented PGE2-mediated M2 MΦ polarization. This phenotype was also supported by observations in the miR-21−/− mouse, which display a higher ratio M2 to M1 MΦs than their WT counterparts. These findings are similar in effect to our own *in vitro* work on miR-24, which used IL-4 and IL-13 as the stimulus for alternative activation, and also demonstrated an M2 bias when its expression was enforced (13). Although additional reports on the role miRNA plays in PGE2 signaling in MΦs are scarce, the case for such a role has been highlighted by the recent finding that miR-155—one of the most extensively studied pro-inflammatory miRNAs (97)—is a component of the PGE2 MΦ response (98).

### Adenosine

Extracellular adenosine is regulated by the nucleoside transporter, adenosine deaminases (ADA) and adenosine kinase. A PubMed search (“microRNA + adenosine + MΦ”) only identified 13 publications; however, the importance of adenosine signaling in not only generating M2 MΦs but also skewing pro-M1 stimuli toward the M2 phenotype (99), suggests that this is an area ripe for miRNA-based study. The current data are extremely limited, but a few miRNAs have already been identified.

Studies of pig retinal microglia and human MΦs revealed ADA2—which lowers extracellular adenosine levels—to be a target of miR-146b (100). Enforced expression of miR-146b in human MΦs suppressed ADA2 expression and inhibited the release of pro-inflammatory cytokines. miRNA may also regulate MΦ activation by targeting adenosine receptors. For example, miR-16 has been reported to target the A2a adenosine receptor (101). One group with a background of investigating adenosine signaling is currently studying the role of miRNA in M2 MΦs (102). They have identified miR-483, miR-877, miR-337-5p, miR-546, and miR-494 as being upregulated, and miR-770-5p, miR-487b, miR-220, miR-212, and miR-712 as downregulated by adenosine signaling in MΦs. It will be interesting to see the fruits of these studies.

## MΦ Suppression

A variety of stimuli can render MΦs resistant to activation or suppress the function of previously activated MΦs. These suppressive stimuli possess common and divergent effects with regard to the resultant MΦ phenotype. For example, while IL-10 may broadly suppress the pro-inflammatory activation of MΦs, the ability of TGF-β to mediate the same effect is similar but less potent and more dependent upon context. MΦ deactivation is also a phenomenon that can occur before or after activation and may be conferred by intrinsic or extrinsic means. For example, IL-10 acts in an autocrine fashion to self-limit the production of pro-inflammatory cytokines by MΦs.

### IL-10

miR-98 is involved in regulating MΦ IL-10 production. Its expression is downregulated in response to LPS, and when its expression is enforced IL-10, production is limited (103). This was reportedly due to direct regulation of the IL-10 transcript, with the authors providing evidence that this contributes to the fine-tuning of endotoxin tolerance. Another miRNA confirmed as a direct regulator of IL-10 is miR-27a (104). Xie et al. reported that this miRNA is downregulated in MΦs activated with a variety of TLR4/TLR2 ligands. When its expression was upregulated, the expression of pro-inflammatory cytokines increased; and when its expression was downregulated, cytokine production increased.

Pro-inflammatory signals other than TLR ligands are also capable of inhibiting IL-10 by altering miRNA expression. Type I IFN inhibits MΦ IL-10 production in an indirect fashion by downregulating miR-145 expression (105). Here, miR-145 was shown to directly target the IL10 gene silencer histone deacetylase 11. Working in opposition to these are those miRNAs which, *via* less canonical mechanisms of action, upregulate IL-10 expression. For example, miR-446I can compete with tristetraprolin (a well-known RNA-binding protein capable of triggering transcript degradation) for a binding site on the IL-10 3′ UTR. miR-446I binding does not mediate silencing or degradation of IL-10 mRNA, but rather enhances IL-10 expression by preventing tristetraprolin-induced degradation (106).

IL-10 is not just a passive target of miRNA regulation. IL-10 also alters MΦ miRNA expression to inhibit various pro-inflammatory molecules both directly and indirectly. For example, the TLR4 signaling pathway is negatively regulated by the IL-10-induced expression of miR-146b (107). This is also a prime example of a single miRNA modulating multiple targets in a coordinated fashion. As previously mentioned, miR-146b targets not only TLR4 but also MyD88, IRAK-1, and TRAF6, resulting in reduced expression of a number of pro-inflammatory cytokines and chemokines. Another way in which IL-10 suppresses the M1 MΦ is by interfering with the normal processing of the pro-inflammatory miRNA, miR-155. IL-10 signaling destabilizes the pri-miR-155 and pre-miR-155 transcripts and also inhibits the final maturation step *via* a mechanism involving STAT3 and SHIP1.

### Corticosteroids

miR-155 is also involved in the suppression of M1 MΦs by corticosteroids (108). miR-155 was identified by Zheng et al. as being downregulated in LPS-stimulated MΦs following glucocorticoid exposure. This was followed by experiments where enforced expression of miR-155 reversed the suppressive effect of glucocorticoid treatment. Despite this, the link between PGE2 and miRNA *in vivo* has yet to be established. Indeed, a miRNA profiling study (109) of the anti-inflammatory effects of corticosteroids in the lung failed to identify any significant changes despite the fact that MΦs are abundant in the respiratory tract even in the absence of infection (110). Still, it remains likely that a more detailed examination of the MΦ population in isolation would identify miRNA changes, as whole-tissue profiling always includes the possibility of false negatives arising from the opposite, but equal, changes in two or more cell-types simultaneously.

### The TAM: Combining M2 and Deactivation

Tumor-associated Mφs combine the characteristics of the M2 MΦ with those of deactivation. Their existence and importance in tumor survival have been known for decades. It was not long after their identification that their generation and phenotypic characteristics were noted to be highly dependent on the tumor microenvironment. Indeed, certain functional characteristics—for example, their dysfunctional processing of tumor antigens, are dependent upon cell-to-cell contact. In this example, contact with malignant cells results in defective phago-lysosomal interactions, which disrupts their capacity to correctly process antigen. This mirrors data published by our group (63) demonstrating how the expression of a single miRNA can result in dysregulated antigen uptake, processing and presentation by MΦs. Given the sensitivity displayed by miRNAs to a wide range of environmental stimuli, it seems likely that altered miRNA expression contributes to the generation of TAMs.

Tumor-associated Mφs are known to reduce patient survival rates by stimulating angiogenesis, tumor cell migration, and metastasis. The promotion of angiogenesis is one of the key functional properties of M2 MΦs, and the growth of new blood vessels not only fuels tumor growth but also provides additional routes by which tumor cells may migrate and metastasize. As tumor growth progresses, the TAMs also produce greater quantities of the M2-biasing differentiation factor, M-CSF, and the M2 promoting cytokine, TGF-β (111). At this stage, TAMs also produce greater quantities of the potent deactivating cytokine, IL-10 (111). Several studies have identified miRNAs involved in the generation, phenotype or function of TAMs. Squadrito et al. have shown that miR-511-3p, which targets the MΦ mannose receptor, is upregulated in TAMs and this is associated with changes in blood vessel morphology (112). miR-125a is also known to modulate TAM differentiation and function. Through targeting of FIH and IRF4, miR-125a has been reported to promote M1 and suppress M2 phenotype (113). Likewise, Lin et al. demonstrated the miR-130a targets PPARγ, a key regulator of immune suppression mechanism, and exhibit antagonistic expression with another M2 Mφ marker, CD163 (114). An *in vivo* study by Xu et al. (115) showed that overexpression of miR-142-3p in M2 MΦ induced selective modulation of transforming growth factor beta receptor 1, which led to subsequent preferential apoptosis in the M2 subset. The previously mentioned studies by Li et al. (57) and Ma et al. (85) also constitute data regarding TAMs. TAM-focused studies such as these set important precedents for what will hopefully become a novel miRNA-based therapeutic strategy for treating cancer.

## Plasticity

The mature MΦ is arguably the most functionally diverse type of leukocyte present in the human body, not only by virtue of the diverse range of roles it fulfils but also in terms of its ability to dynamically switch its functionality in response to changing stimuli. This plasticity is readily apparent in the context of M1 vs. M2 activation, where the sequential substitution of one set of stimuli for the other results in rapid changes at the level of gene transcription, miRNA regulation, and protein expression. In light of MΦ plasticity, it is tempting to assume that any miRNA that regulates the response to classical or alternative, or indeed innate, signaling also regulates plasticity. The majority of published articles describing miRNA expression in the context of MΦ plasticity have focused on their positive or negative involvement in the M1 or M2 polarized states, with only a handful having included data where polarizing stimuli are reversed (13, 81).

We have studied the capacity of miR-24 to regulate MΦ plasticity in the context of the interaction between the host (MΦ), pathogen (bacteria), and environment (cigarette smoke) (12). Our previous studies had focused on the role played by miR-24 in MΦ activation and had revealed it to be a negative regulator of TLR-mediated pro-inflammatory cytokine production (11, 13). In a related study, we had also observed differences in MΦ activation between LPS isolated from a periopathic bacterium cultured in the absence or presence of cigarette smoke extract (CSE) (21). Smoking is an environmental factor known to alter the structure and immunogenicity of LPS (113) as well as inflammatory responses within the oral cavity (114). Counterintuitively, the bacteria that drive the chronic inflammatory state in periodontitis typically elicit weaker pro-inflammatory responses than their non-periopathic neighbors. This can be rationalized when thought of as a factor promoting the survival of these periopathic bacteria under normal conditions (i.e., in the absence of periodontitis). This study not only supports a role for miR-24 in regulating the transition between M1 and M2 states, but also differential miR-24 expression as a route through which environmental changes are translated into changes in MΦ function.

In these experiments, M1 MΦs were generated using four sets of pro-inflammatory stimuli: IFN-γ, a combination of IFN-γ plus TNF-α, IFN-γ plus TNF-β, or IFN-γ plus IL-17. M2 MΦs were generated using IL-4 plus IL-13. M1 MΦs generated in this fashion produced higher quantities of pro-inflammatory cytokines than their unactivated counterparts when stimulated with periopathic LPS. Enforced expression of miR-24 in MΦs reduced the observed enhancement of pro-inflammatory cytokine production in M1 MΦs. At the same time, enforced expression of miR-24 enhanced the ability of IL-4 and IL-13 to generate M2 MΦs. Furthermore, when these M2 MΦs were exposed to M1-inducing stimuli, they resisted conversion. These modifications in M1/M2 polarization and plasticity were not complete, which is to say that not all M1/M2-associated changes were affected by the enforced expression of miR-24. For example, miR-24 inhibited LPS-induced TNF-α either completely, partially, or not at all, depending on the particular set of cytokines used for M1-induction.

Returning to the environmental component of this study, it was noted that while miR-24 expression is downregulated by LPS—an observation that is consistent with the downregulation of many other anti-inflammatory molecules by this stimulus—it was not observed when the CSE-modified version was used. We postulate that the maintenance of miR-24 expression with CSE-modified LPS likely contributes to its reduced inflammatory potential. It is tempting to postulate that similar environmental modifications in the immunogenicity of local bacterial flora *via* altered miRNA expression may contribute to other diseases where disturbances in MΦ activation confer pathology. For example, alterations in the composition of gut flora through changes in diet or use of antibiotics/probiotics alter the susceptibility of mice to EAE (115). While this MΦ-specific link between bacterial flora and altered miRNA is largely speculative, there are a number of reports of dysregulated miRNA expression in the EAE model—as well as from MS patients. We emphasize the potential contribution of environmental-mediated alterations in miRNA-regulation of MΦ polarization and plasticity as changes in lifestyle (for example, exposure to environmental toxins) could inform screening protocols where miRNAs are the disease biomarkers (116).

miR-223 is a multi-functional miRNA that has recently been shown to promote plasticity from the M1 to M2 phenotype, as evidenced by a decrease in M1 markers (such as iNOS-2), increase in M2 markers (such as Arg-1) and reduced production of pro-inflammatory cytokines (117). The regulatory properties of miR-124—discussed earlier in the context of M2 activation—extend to plasticity. This property is mediated by C/EBP-α; however, how this molecule suppresses M2 polarization in the context of EAE remains to be seen. Like many of the plasticity-associated miRNAs that have been reported, our understanding of their regulatory capacity is severely limited. While it is tempting to simply apply our knowledge of polarizing miRNAs to plasticity, this must be supported by empirical data truly demonstrating such a role—certainly *in vitro* and preferably *in vivo*. One hopes that if this review were to be written even a few years from now, it would see the movement of many of the M1/M2 polarizing miRNAs into this section.

Banerjee et al. have reported on let-7c expression and function during the re-polarization of M1 to M2 MΦs and vice versa (19). The authors reported that the expression of let-7c was higher in newly generated M2 MΦs and that its expression tracks with plasticity. It was downregulated during M2 to M1 conversion and upregulated during M1 to M2 conversion. Furthermore, its expression in M2 MΦs was also downregulated by LPS stimulation. In a finding similar to our own description of miR-24, the authors also found that enforced expression of let-7c diminished the M1 phenotype while promoting M2 polarization. While our own studies indicate p110δ as being the mediator of miR-24-mediated M1/M2 regulation, Banerjee et al. identified C/EBP-δ as the target of let-7c. The phenomenon of different miRNAs converging at the level of phenotype regulation while diverging at the mechanistic level is not an uncommon one, and further suggests that a multi-miRNA approach to miRNA-based therapies may be advantageous to prevent loss of efficacy due to redundancy and/or to achieve a phenotypic profile better tailored to the treatment of specific disease parameters. This approach would need to be based on a thorough understanding of how each miRNA functions alone and with others at the level of coordinated network regulation.

## APC Functionality and Th Cell Polarization

Much of the research looking at miRNA function in the context of adaptive immunity has focused on the productive activation of lymphocytes by DC. The connection between MΦ APC functionality and polarization/plasticity is not as readily apparent as it is for say, pathogen recognition; however, direct and indirect links do exist. Clearly, the type of Th cell generated by the interaction between APC and naive CD4+ T cell is going to impact MΦ polarization. Th1-dominant responses promote M1 MΦ polarization *via* increased IFN-γ and TNF-α levels, Th2-dominant responses promote M2 MΦ polarization *via* IL-4, while Treg-dominant responses promote MΦ deactivation *via* IL-10. This forms a positive feedback loop for MΦ polarization, as M1 MΦs are more likely to support Th1 differentiation (*via* IL-12 secretion) and M2 MΦs are more likely to support Th2 differentiation (*via* IL-4 secretion). The same principle can be applied to the plasticity of MΦs, particularly at the population level, as an inflammatory environment that is shifting from one that is rich in pro-inflammatory signals to one that is rich in anti-inflammatory signals will be accompanied by an M1 to M2/deactivation transition.

Recent advances in our understanding of the signaling events at the immunological synapse have changed the perception of this process from being one that is unidirectional with regard to its signaling to one that is bidirectional. For example, CD40 ligation has been shown to activate MΦs *via* a mechanism that includes endogenous IFN-γ production and this depends upon the presence of IL-12 (118). Several recent reports have highlighted a role for miRNA during such APC-lymphocyte interactions. Most of these have used DC rather than MΦs, and while these data do not automatically apply to MΦs, a combination of common progenitor, common functionality, and instances where miRNA function has been shown to be comparable in DC and MΦs, lends weight to the likelihood that at least some of these instances of miRNA regulation are also present in MΦs. This may not be focused at the level of MΦ-naive lymphocyte interactions within secondary lymphoid tissue (the *in vivo* relevance of which remains a topic of debate), but are likely to be relevant to the interactions that occur at the site of infection/inflammation. These interactions are posited to provide a source of antigen-specific survival signals to lymphocytes, but would equally present an opportunity for lymphocyte modification of MΦ polarization activation state.

Our own investigations into miRNA-mediated regulation of APC functionality have focused on the enforced expression of miR-24/30b/142-3p in human monocyte-derived APC (MΦ/DC)—T cell co-cultures (63). Here, the immediate effect of enforced miRNA expression is a reduction in the ability of APC to take up antigen. This is accompanied by reduced antigen-processing and proteolytic degradation. Furthermore, this was observed in both human and murine APC. This allowed us to explore downstream effects on T cell proliferation and differentiation using a single antigen system. Using APC and T cells obtained from OT-II mice, which possess a TCR that is specific only for OVA antigen, we discovered that T cell proliferation was suppressed when the expression of any of these three miRNAs was enforced in the APC. Furthermore, this was accompanied by a reduction in Th1/17-associated cytokines.

Intriguingly, we observed a specific reduction in the Th1-associated cytokines IFN-γ and TNF-α, but not the Th2-associated cytokine IL-4. These experiments raise two important points with regard to the therapeutic application of these miRNAs. First, it builds upon our earlier work describing the inhibitory effects of miRNAs on M1 MΦ polarization to include downstream effects on adaptive immunity. Second, the reduced production of pro-inflammatory cytokines in these coculture systems was not due to decreased APC secretion, but was a T cell-dependent phenomenon, indicating that the manipulation of APC miRNA expression can have both immediate and long-lasting effects. Taken together, our studies on miR-24, miR-30b, and miR-142-3p may provide a route toward novel therapies aimed at treating chronic inflammatory disorders.

## Therapeutic Potential

The therapeutic potential of using miRNA mimics/inhibitors to modulate MΦ polarization and plasticity is undeniable. However, enthusiasm is tempered by a number of practical issues. These include finding a suitable method of delivery, the issue of systemic vs. tissue/cell type-specific delivery, and the potential for off-target effects. These are not insurmountable problems and progress has been—and continues to be—made. Ultimately, the nuances in phenotype skewing meted by specific miRNAs will ideally, be tailored to the specific clinical parameters of each disease. The knowledge base required to identify these miRNA-disease pairings already exists within the current body of published literature describing miRNA modulation of MΦ polarization, plasticity, and function.

Many of the commonly used *in vitro* techniques for introducing miRNA mimics or inhibitors into cells are not suitable, at least in their current state, for clinical use. The reasons for this include considerations such as *in vivo* stability, transfection reagent toxicity, lack of cell type/tissue-specific targeting, or unacceptable gaps in our knowledge when it comes to the list of direct targets of any given miRNA. These issues continue to be addressed at the level of basic and translational research.

To utilize miRNA as therapeutics, either miRNA or its antisense sequence can be used. Antisense oligos (ASOs) were used as synthetic miRNAs to target mRNA of therapeutic value, while antimiRs (miRNA inhibitors) can bind mature miRNA and block their post-transcriptional activity (119–121). A key obstacle for RNA-based therapeutics is their susceptibility to endogenous RNase activity. Various approaches have been developed to address the issue of miRNA stability for *in vivo* studies. Enzyme-resistant biochemical modifications of synthetic RNA molecules were found to enhance stability of miRNA targeting molecules from the degradation by serum or intracellular RNases (119, 120). For instance, non-binding oxygen in ASOs were replaced with sulfur to generate phosphorotiorate nucleotides. Furthermore, introduction of 2′-*O*-methyl groups rendered improved nuclease resistant and increased binding affinity to target miRNA, thus enhancing sequence specificity. In yet another approach, 2′-oxygen and 4′-carbon on ribonucleotide backbone were chemically locked (120). These modified oligonucleotides, termed locked nucleic acids (LNA), have been very successful in studying *in vivo* and *in vitro* miRNA functions. Indeed, LNA modified miR-122 inhibitor (a clinically promising cellular miRNA targeting hepatitis C virus) has shown improved clinical outcomes. Blocking miR-122, a host miRNA required for HCV replication, reduced viral titers in animal studies and is currently under phase two clinical trial (122, 123). Table 3 lists examples of miRNAs that are currently undergoing phase I/II clinical trials with indicated chemical modifications. These candidate therapeutic miRNAs were selected from the European Union Clinical Trials (https://www.clinicaltrialsregister.eu/ctr-search/search?query=microRNA).

**Table 3:** Overview of current therapeutic trials utilizing miRNAs.

Name	Targeted miRNA	Target diseases	Technology	Mechanism	Stage	ClinicalTrials.gov identifiers
Mirna Therapeutics	miR-34	Primary liver cancer or solid cancers with liver involvement	Mimic LNPs (Smarticles)	Tumor regression, enhanced survival and inhibited the growth of non-hepatic tumors	Phase 1, completed	NCT01829971

Mirvirasen (Santaris Pharma A/S and Hoffmann-La Roche)	miR-122	Hepatitis C	Anti-miR LNA-modified antisense inhibitor delivery system	Reduction in viral plasma RNA levels compared from baseline	Phase 2a	NCT02031133

MRG-201 (MiRagen Therapeutics)	miR-29	Scleroderma	Mimic Cholesterol-conjugated miRNA duplex	Reduction in aberrant cell proliferation	Phase 1	NCT02603224

RG-125/AZD4076 (Regulus Therapeutics)	miR-103/107	Type 2 diabetes, non-alcoholic fatty liver diseases	AntimiR GalNAc-conjugated		Phase I/IIa, ongoing	NCT02826525

MRG-106 (miRagen Therapeutics)	miR-155	Cutaneous T cell lymphoma and mycosis fungoides	AntimiR LNA-modified antisense inhibitor		Phase 1	NCT02580552

miRagen Therapeutics	miR-92	Pheripheral artery disease		Improves recovery of damaged tissue, enhance blood vessel growth	Pre-clinical	–

Another challenge in using miRNA-based therapeutics is their targeted delivery. Several different approaches viz., liposomes, dendrimers, cholesterol conjugation, polyethylenimine (PEI), and pH-based peptide are shown as promising vehicles to achieve efficient miRNA delivery (119, 120, 122). Nonetheless, based on the target tissue heterogeneity, its location, cytotoxicity, the ribo-oligo delivery may be hampered. Therefore, employing more than one delivery systems or screening of various vehicles to monitor tissue-specific efficiency may prove beneficial.

The use of nanoparticles as delivery vectors appears promising. These can be formed from various molecules, and when combined with certain modifications, may be chosen on a case-by-case basis to target specific cell types. For example, Tran et al. recently demonstrated an MΦ targeted approach that allowed for the delivery of miR-223. MΦ targeting was achieved by using CD44-targeting hyaluronic acid-poly (ethyleneimine) (HA-PEI) nanoparticles. Interestingly, while this was achievable with a miR-223 cargo composed of either miR-223 duplexes or a miR-223 encoding plasmid, the duplexes resulted in the greatest miR-223 expression. Importantly, the authors also demonstrated therapeutic proof-of-principle as this therapeutic strategy reduced LPS-induced inflammation *in vivo*.

The therapeutic potential of miR-27a has been investigated in the context of treating pathology associated with alcohol abuse and hepatitis C. Both of these insults are associated with liver disease (as characterized by inflammation, hepatitis, or cirrhosis) and both enhance miR-27a expression in monocytes and monocyte-derived MΦ (124). It will be interesting to see whether the M2-promoting capacity of miR-27a inhibitor translates to *in vivo* benefit. Silencing miR-155 expression may also prove to be of clinical benefit. MΦs from miR-155 KO mice are skewed toward the M2 phenotype and these mice are more resistant to ischemia–reperfusion injury (IRI) than their WT counterparts (125). This attenuation is associated with reduced production of TNF-α, IL-6, and IL-12p40, which suggests that our own findings of miR-24/30b/142-3p mediated suppression of these cytokines may be relevant to IRI (11, 13), and by extension, the hemorrhagic and septic shock that causes IRI. Furthermore, an additional component of the attenuated IRI in these miR-155 KO mice was a reduction in Th17 differentiation and IL-17 production—another property we have described for miR-24/30b/142-3p (63). Our work in this area was focused on enforced expression of these miRNAs in APC alone, and when considered alongside our description of their M2-promoting/M1-suppressing properties encourages further investigation of their therapeutic properties.

Autoimmune diseases are a prime candidate for miRNA-based therapies. These include pathologies mediated by the pro-inflammatory response of both the innate and adaptive arms of the immune system. A significant portion of the current body of miRNA literature describes miRNA regulation of inflammation, a significant subset of which covers the activation of myeloid inflammatory cells. As MΦs are key mediators of inflammation and immunopathology in many autoimmune diseases, modulating MΦ polarization and plasticity *via* miRNA manipulation is a promising therapeutic strategy. miR-29 is a miRNA that has been shown by Salama et al. to modulate both innate and antigen-specific immune responses in an adoptive transfer model of autoimmune diabetes (126). This particular study did not target MΦs specifically, but it does highlight the ability of miRNA-based therapeutic strategies to suppress both innate and adaptive immunopathology.

Studies on pathogens and immune-related diseases have shed light on our understanding of MΦ polarization in context of therapeutics. For instance, *M. tuberculosis* infection in MΦ alters expression of several miRNAs (41–43). In particular, induced levels of miR-26a and miR-132 suppress IFN-γ signaling *via* direct targeting of p300 (32). Interestingly, different strains of mycobacteria can selectively influence the phenotype of MΦ (Figure 2). While *M. tuberculosis* and *Mycobacterium smegmatis* upregulate miR-142-3p leading to N-wasp downregulation and reduced phagocytosis (inhibition of M1 phenotype), *M. bovis* downregulates the same miRNA consequently activating NF-κB (M1 phenotype) (45, 127). This suggests that pathogens modulate MΦ polarization for their survival and, thus, it provides a novel approach to target MΦ phenotype to expose pathogens to a suitable phenotype.

Exosome-based strategy to deliver miRNAs is embraced as novel, non-immunogenic, broader, or cell-specific miRNA delivery methods. These membrane enclosed miRNA/mRNA/protein containing endosome-derived nanovesicles are ubiquitously secreted by cells (128, 129). A role of exosomes in drug delivery has been proposed (130, 131). Indeed, MΦ-derived exosomes has been demonstrated to cross blood–brain barriers and deliver brain-derived neurotrophic factor (BDNF) (132). Thus, MΦ-derived exosomes can be considered for the treatment of brain-related disorders. Employing this strategy to package-specific miRNA with MΦ modulatory potential should be examined (Figure 2). Indeed, exosome-mediated uptake of let-7b, an IL-6 targeting miRNA, by tumor MΦ has been shown to skew phenotype toward M2 MΦ (57). Together, these examples highlight new alternative approaches that may provide better and efficient ways to evaluate the clinical potential of miRNAs.

The previous examples have focused on miRNA suppression of inflammation and immunopathology to prevent the cell death and tissue damage caused by disease. miRNA-based therapies may also be employed to actively promote recovery after the damage has been done—a minor, but important, distinction. Guo et al. recently provided proof of principle for how *in vivo* manipulation of miRNA expression can enhance the recovery phase of disease. Here, they combined a murine model of acute lung injury and treatment with a miR-155 inhibitor (133). Enhanced recovery in this model involved the expansion of Tregs and the M2 MΦ population.

The induction and resolution of disease are clearly intimately connected. MΦ, by virtue of being long-lived and capable of plasticity, may be envisioned as bridging either end of the disease spectrum. Experimental manipulation of miRNA expression in MΦ for the purposes of modulating their dynamism and plasticity, therefore, has the potential to deliver clinical benefit across opposite ends of the inflammatory, immunological, and pathological spectrums.

## Concluding Remarks

Significant progress has been made in identifying the role of miRNA in regulating MΦ polarization and plasticity. This progress builds upon many decades of work seeking to understand how gene expression is regulated. The addition of miRNA to this regulatory machinery has expanded our understanding of how MΦ are able to respond to external stimuli in such a dynamic fashion—stimuli that are often contradictory to those it has only recently received. From what we know thus far, miRNA represents an important mechanism for altering MΦ function without the requirement for changes in gene transcription. Identifying the compete repertoire of direct miRNA targets in different cell types and diseased tissues will prove extremely valuable in employing miRNA therapeutics with high confidence. Furthermore, the multi-functional role of MΦ in initiating and resolving inflammation makes it a very attractive therapeutic target for many types of disease. The clinical application of miRNA is tantalizingly close. Continued progress in the identification of miRNAs—along with descriptions of their complex regulatory properties, both in the context of disease and MΦ function, brings us ever closer to the dawn of a new class of therapeutic agents.

## Author Contributions

All of the authors made substantial contributions to the conception and design of the article. All authors contributed to the initial draft and subsequent revisions. All authors provide final approval for the article to be published. All authors agree to be accountable for all aspects of the work, including ensuring that the content is accurate.

## Conflict of Interest Statement

The authors declare that there is no conflict of interest, be it financial, commercial, or other.
